# The functional role of spatial anisotropies in ensemble perception

**DOI:** 10.1186/s12915-024-01822-3

**Published:** 2024-02-05

**Authors:** Natalia A. Tiurina, Yuri A. Markov, David Whitney, David Pascucci

**Affiliations:** 1 Laboratory of Psychophysics, Brain Mind Institute, École Polytechnique Fédérale de Lausanne (EPFL), Lausanne, Switzerland; 2 Department of Psychology, Technische Universität Dresden, Dresden, Germany; 3 Department of Psychology, Goethe University Frankfurt, Frankfurt am Main, Germany; 4 Vision Science Graduate Group, University of California, Berkeley, Berkeley USA; 5 Department of Psychology, University of California, Berkeley, Berkeley USA; 6 Helen Wills Neuroscience Institute, University of California, Berkeley, Berkeley USA

**Keywords:** Ensemble perception, Ensemble coding, Summary statistics, Statistical perception, Visual uncertainty, Spatial biases

## Abstract

**Background:**

The human brain can rapidly represent sets of similar stimuli by their ensemble summary statistics, like the average orientation or size. Classic models assume that ensemble statistics are computed by integrating all elements with equal weight. Challenging this view, here, we show that ensemble statistics are estimated by combining parafoveal and foveal statistics in proportion to their reliability. In a series of experiments, observers reproduced the average orientation of an ensemble of stimuli under varying levels of visual uncertainty.

**Results:**

Ensemble statistics were affected by multiple spatial biases, in particular, a strong and persistent bias towards the center of the visual field. This bias, evident in the majority of subjects and in all experiments, scaled with uncertainty: the higher the uncertainty in the ensemble statistics, the larger the bias towards the element shown at the fovea.

**Conclusion:**

Our findings indicate that ensemble perception cannot be explained by simple uniform pooling. The visual system weights information anisotropically from both the parafovea and the fovea, taking the intrinsic spatial anisotropies of vision into account to compensate for visual uncertainty.

**Supplementary Information:**

The online version contains supplementary material available at 10.1186/s12915-024-01822-3.

## Background

**Fig. 1:**
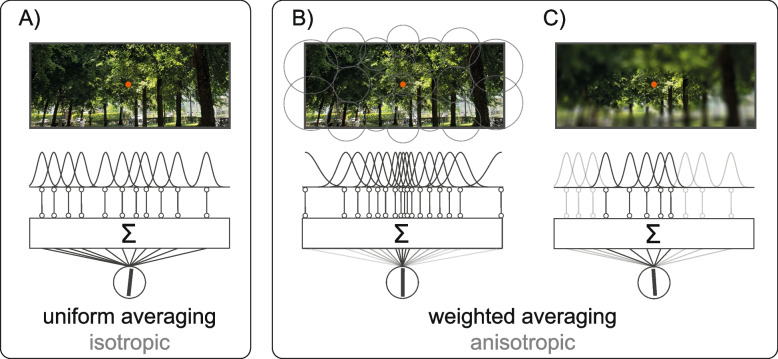
Ensemble coding in natural scenes. Example of isotropic (uniform) and weighted (anisotropic) averaging in a simple pooling scheme. **A** Uniform: local features (e.g., the orientations of the trees) are pooled together over the entire visual field, independently of their location and distance from the fovea (the orange fixation spot). In uniform pooling, the activity of many neurons encoding local features (depicted as a set of Gaussian tuning functions) is equally weighted and integrated over large areas of space. Integration (Σ) results in a single estimate: the average orientation of all the trees. **B** Weighted averaging: integration occurs in anisotropic space, due to changes in the density and size of receptive fields (illustrated by gray circles). In this case, ensemble coding is biased towards features at the center of the scene, because of the higher number and narrower tuning of neurons at the fovea. Hence, much more information is received from the central visual field. **C** Anisotropies due to spatial biases (here, a bias towards the center and the left-hand side is illustrated by the difference in blurring between the central and left-hand region and the rest of the image). Features inside the region of preferential processing (black tuning functions and lines) are weighted more than features outside (gray tuning functions and lines). Note that the three scenarios can lead to different average estimates (the oriented line resulting from integration)

The visual world is extremely rich and complex. Yet, much of the information it contains is redundant and can be easily compressed without loss, imagine a natural scene, like a park full of trees (Fig. 1). Despite the huge number of details, many features look alike. It takes only a split-second to recognize the group of trees, their average color, orientation, or even typology, without inspecting the scene in detail. This process is known as ensemble perception [1–3].

In ensemble perception, the brain combines information from multiple stimuli to represent their *ensemble statistics*, like the average and variability [1–3]. It is believed that ensemble statistics are extracted within a few milliseconds and beyond the bottleneck of attention and single-object recognition [2, 4–9]. The rapid extraction of ensemble statistics supports fundamental visual functions, such as gist perception [10, 11], grouping [12–16], and visual search [17–19], but the underlying mechanisms are still debated [3, 20]. In particular, it remains unclear how the visual system integrates information from local elements dispersed over the entire visual field.

One prevailing view argues that ensemble statistics are extracted via mechanisms that operate in parallel treating all elements the same [6, 21, 22]. For example, in one class of models, ensemble statistics are computed by pooling over many local features that are initially processed in parallel (Fig. 1) [23–26], but see [27–30]. Models of this kind rest on the implicit assumption that ensemble perception operates in a spatially uniform field—i.e., that elements at the fovea or far in the periphery contribute equally to ensemble perception (Fig. 1A).

There are several reasons to question this assumption. Firstly, spatial vision is not uniform. Visual resolution is higher at the fovea (Fig. 1B) [31–33], there is receptive field scaling [34–36] and well-known left–right and up-down asymmetries (Fig. 1C) [37–40]. Secondly, several studies have reported systematic biases in ensemble statistics due to factors such as the temporal order of element presentation [41] and the spatial location [42–44], suggesting that the underlying mechanisms may indeed operate non-uniformly in space and time.

However, not all known anisotropies in visual processing are evident in ensemble perception, but rather some specific ones. For example, upper and lower visual field anisotropies, which strongly affect phenomena like visual crowding, do not seem to affect ensemble perception [45]. Instead, there is consistent evidence of systematic biases towards the center and left-hand side of the visual field, with foveal regions receiving more weight than parafoveal and peripheral ones [43, 44, 46, 47]. Several studies have proposed that such spatial biases, common in many other domains of vision research [48–52], might be beneficial for ensemble perception [43, 44, 46, 47], but their nature remains largely unknown. For instance, it is unclear whether spatial biases reflect idiosyncratic aspects of how the visual system processes information across the visual field or efficient strategies to combine information from more reliable regions.

Here, we hypothesize that spatial biases serve as adaptive and flexible mechanisms to optimize ensemble perception. We reasoned that, if spatial biases reflect a way to optimize ensemble perception, rather than being idiosyncratic and invariant aspects, they should become more evident and advantageous under noisy and uncertain sensory input.

**Fig. 2:**
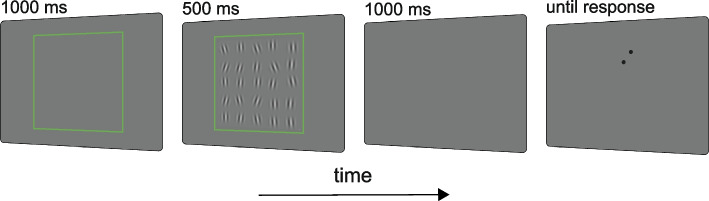
Schematic of the sequence of events on each trial. The green frame indicated the beginning of the trial and the region of the display where both the ensemble and the response tool were presented. An ensemble of oriented Gabor patches was then shown for 500 ms, followed by a blank interval before the response. The response tool appeared at random locations

To test this idea, we performed a series of experiments where observers reproduced the average orientation of an ensemble of Gabors (Fig. 2). We manipulated the uncertainty associated with the ensemble through (1) the oblique effect (experiment 1), (2) the ensemble variability (experiment 2), (3) the ensemble duration (experiment 3), and (4) the variability in the temporal order of individual elements (experiment 4). Using a spatial weighted average model [43, 53], we reconstructed the spatial profile with which observers integrated elements at different locations. We tested the observed spatial distribution of weights against the hypothesis of a uniform field. In line with prior work, we found strong spatial anisotropies, evident across all experiments. These anisotropies arose from potentially independent spatial biases: a robust and stable bias towards the center of the visual field and a leftward and upward bias towards locations near the center. The central bias, observed in all experiments, scaled with the ensemble uncertainty but persisted under different durations of the ensemble, suggesting a hard-wired mechanism that may play a compensatory role in ensemble perception.

Overall, our findings reveal a strong anisotropic spatial field within the parafoveal region that is functional to ensemble perception and neglected by current models based on simple uniform pooling and population responses. We propose that the visual system combines noisy information from the parafovea with more reliable estimates at the fovea to compute ensemble statistics. This weighted integration is a stable aspect of ensemble perception, which compensates for visual noise and uncertainty.

## Results

### Experiment 1

#### Spatial anisotropies in orientation ensemble statistics

We used a continuous report, method of adjustment to investigate spatial anisotropies in orientation ensemble perception. Observers were presented with 25 oriented Gabor patches, spaced within a square region, and reproduced the perceived average orientation by adjusting a response tool (see Fig. 2 and the “Methods” section). Using a spatial weighted average model, we estimated the weight assigned to each Gabor at each location in the process of estimating the ensemble statistics [43, 53]. Weights were computed at the individual level as coefficients of linear regression, transformed into *t*-scores before group analysis (see the “Methods” section).

**Fig. 3:**
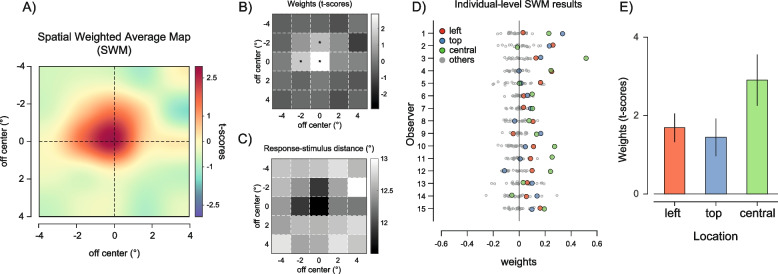
Results of experiment 1. **A** Spatial weighted maps (SWM) represented as interpolated heat maps (see the “Methods” section) to depict the increase in weights (in the colormap from blue to red) at central, leftward, and upward regions. **B** Raw weights from the spatial weighted average model (*t*-scores) in grayscale (white means larger weights). Asterisks indicate locations with weights significantly larger than those expected in uniform averaging (permutation statistics, see the “Methods” section). **C** The response-stimulus distance metric with values in grayscale (black means lower distances between the reported orientation and the orientation of the stimulus at each location). **D** Raw weights for the central (green dots), leftward (red dots), and upward locations (blue dots) compared to weights at all other locations (gray dots) at the individual subject level. **E** Bar plot of the estimated weights at the three significant locations. Error bars are standard errors of the mean (SEM)

The spatial profile of weights (spatial weight maps (SWM)) obtained with this method revealed clear anisotropies: first, a strong bias towards the central element of the ensemble, which was presented at the fovea; second, a bias towards the leftward and upward locations immediately surrounding the center (Fig. 3A). The weights at these locations were significantly larger than those expected from spatially uniform summary statistics (*p*_corr_ < 0.05; Fig. 3B, permutation statistics, see the “Methods” section). At the individual level, the bias towards the central element was consistent and evident in 12 out of 15 observers (Fig. 3D). The effect sizes of the estimated bias against zero were large for the central (Cohen’s *d* [*d′*] = 1.147) and leftward location (*d′* = 1.197) and medium for the upward location (*d′* = 0.775, Fig. 3E). We confirmed these results in a control analysis where we computed a complementary metric, the response-stimulus distance—i.e., the absolute distance in degrees between the reported orientation and the orientation of each Gabor, confirming that observers’ reports were systematically closer to the orientation of the upward, leftward, and central Gabor than they were to the orientation of all the other stimuli (Fig. 3C; upward location: response-stimulus distance = 11.908, *d′* of the difference with all other locations except the central, leftward, and upward =  − 1.028; leftward location: response-stimulus distance = 11.907, *d′* =  − 1.247; central location: response-stimulus distance = 11.523, *d′* =  − 1.132). This pattern, particularly the central and left-side bias, closely resembled the one found for size averaging in previous work [43].

#### The central bias increases with the oblique effect

**Fig. 4:**
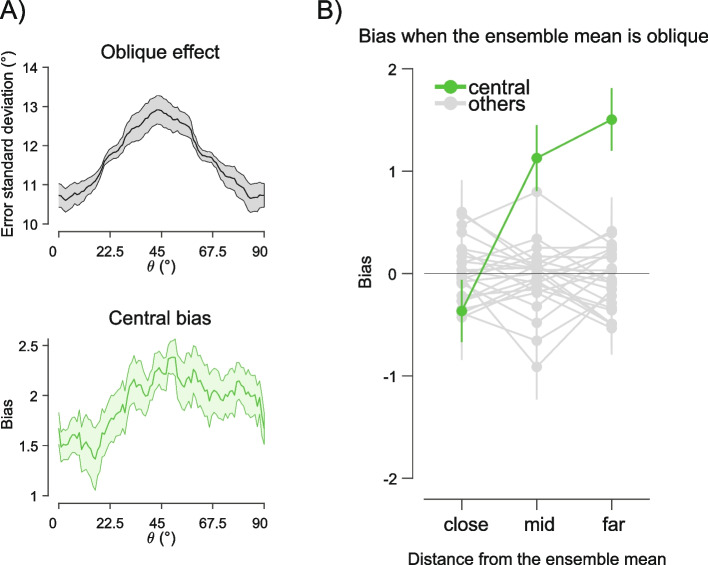
**A** Oblique effect (top plot, gray curve) and central bias (bottom plot, green curve) as a function of the average orientation of the ensemble. The 0–90° range of orientations in the *x*-axis summarizes the effects by collapsing orientations within the actual 0–90° and the 90–180° ranges. Shaded areas are SEM. **B** Bias towards the central vs. other locations in trials where the average ensemble orientation was at and near the obliques. Separate biases are computed as a function of the absolute distance between the orientation of the stimulus at the location of interest and the average orientation of the ensemble (e.g., for “close” distances, the stimulus had an orientation near the oblique; for “far” distances, the stimulus had an orientation away from the oblique)

The precision of orientation estimates typically varies as a function of the stimulus angle, with cardinal orientations represented more precisely than obliques—i.e., the “oblique effect” [54, 55]. We exploited the oblique effect to test whether the observed biases increased when the average orientation of the ensemble was near the obliques and, therefore, more uncertain. To this aim, we estimated multiple SWMs and derived an estimate of the central bias as a function of the average orientation of the ensemble, in sliding widows of 20° within the interval between cardinal axes (e.g., collapsing vertical and horizontal, see the “Methods” section). The central bias was estimated by subtracting the *t*-scored weight for the central location from the average *t*-scored weights at all other locations (Fig. 4A).

When the average orientation was near the obliques, the bias towards the center increased compared to when the average orientation was near the cardinals. This was verified by a significant positive linear slope of the bias from cardinal to obliques (mean slope across subjects = 0.017, *t*(14) = 2.161, *p* = 0.048, *d′* = 0.558). Neither the leftward nor the upward biases showed a similar effect (all slopes were non-significant). The central bias and the oblique effect also exhibited a very similar pattern over the cardinal-to-oblique orientation range, increasing for average orientations near the obliques and decreasing near the cardinals (Fig. 4A). Hence, the anisotropy towards the center scaled with the oblique effect, a typical index of increased uncertainty in orientation processing.

#### Combining foveal and parafoveal elements

**Fig. 5:**
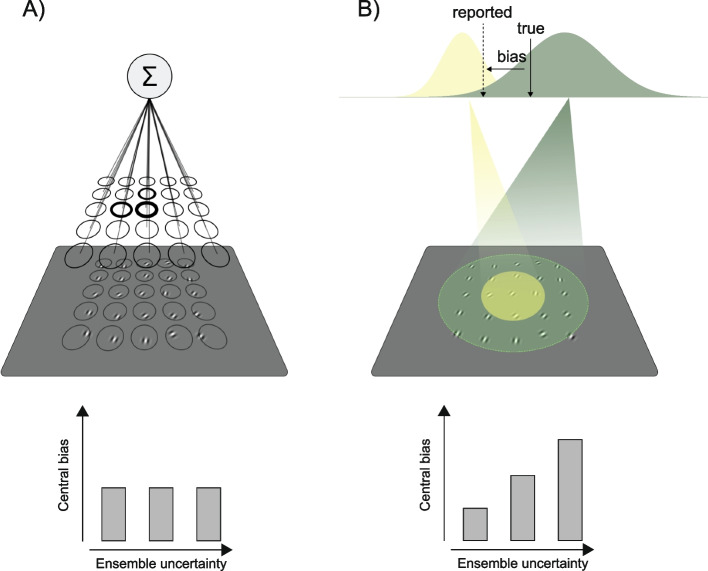
Candidate models of the central bias in ensemble statistics. **A** A non-uniform pooling scheme—i.e., weighted average, in which central elements are given greater integration weights compared to the rest. This model predicts a fixed bias with no changes due to the overall ensemble uncertainty (exemplified in the bottom plot). **B** Observers combine the representation of the stimulus at the fovea with the statistics of the elements in the parafovea. As the uncertainty in the ensemble statistics increases, the estimates in the parafovea become noisier while the representation of the central elements is unchanged: the central bias increases in strength (exemplified in the bottom plot)

The results of our first experiment so far revealed strong spatial anisotropies in ensemble statistics of orientation. A tentative model of such anisotropies can be based on the idea of a non-uniform pooling or “weighted average” of local features, where elements at central locations always receive greater weights compared to the rest (Fig. 5A). Such a model would be in line with many existing simple pooling schemes, with the only exception being the biased distribution of weights during integration. However, the increase of the central bias with the oblique effect cannot be explained by a simple weighted average. Indeed, a systematic and fixed bias in local weights would produce a bias in the final estimate that is fixed and independent of the uncertainty in the ensemble—i.e., even if the central element is weighted two times more than the rest, the bias towards the center remains the same (e.g., two times) no matter the ensemble variability or uncertainty. These results seem to suggest instead that, in extracting the whole ensemble statistics, observers combine the representation of the central element with the noisy average of elements in the parafovea (Fig. 5B). If this combination depends on the relative uncertainty of foveal and peripheral information, with the more reliable source given greater weights, then the bias towards the center should increase as the uncertainty in the parafovea increases, because the central element uncertainty is fixed.

Crucially, this also predicts that when the central estimate is noisier—e.g., because the central stimulus itself is near or at the oblique—the bias decreases. We verified and confirmed this prediction by comparing the central bias as a function of the distance of the central stimulus to the oblique orientation, in trials where the ensemble average orientation was near the obliques (Fig. 4B). This analysis revealed a significant increase in the central bias as the orientation of the central stimulus deviated from the oblique (repeated measures ANOVA with factor “distance” [close, mid, far], *F*(2, 28) = 10.19, *p* < 0.001, ${\eta }_{p}^{2}$ = 0.421). Hence, when the central stimulus itself was oblique, the bias was reduced because the central stimulus was also more uncertain. No similar pattern was found when considering biases at all other locations (gray dots and lines in Fig. 4B).

### Experiments 2 and 3

In experiment 1, we found larger biases towards the center of the visual field when the ensemble average orientation was closer to an oblique angle. This suggested an inverse relationship between the magnitude of the anisotropy and the perceived uncertainty in the stimulus, as oblique orientations are usually more uncertain than cardinal ones [54–56]. To test this relationship more directly, we performed two experiments, in which uncertainty was manipulated by varying the standard deviation of the ensemble orientations (*σ*; experiment 2) and the stimulus duration (experiment 3).

#### Effect of the ensemble variability

**Fig. 6:**
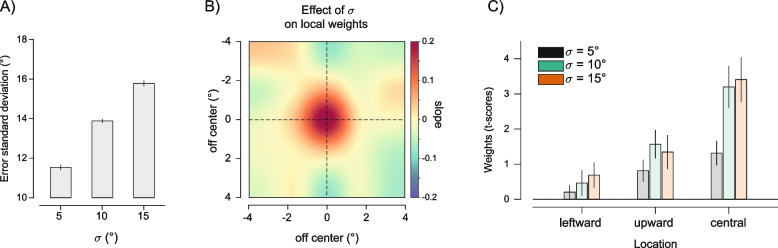
Results of experiment 2. **A** Standard deviation of the errors in adjustment responses as a function of the ensemble variability *σ*. **B** Effect of *σ* on local weight changes, showing the increase in the central bias with the increasing variability in the ensemble’s orientations. **C** Weights (*t*-scores) estimated at each of the three locations of interest (leftward, upward, central), as a function of the ensemble *σ*

In experiment 2, 28 participants reproduced the average orientation of the ensemble at 3 different levels of *σ*: 5°, 10°, and 15°. The standard deviation of adjustment errors increased with the increasing *σ* (Fig. 6A; *F*(2,54) = 263.49, *p* < 0.001, ${\eta }_{p}^{2}$ = 0.907), as expected because of the increasing uncertainty. We extracted the weights (*t*-scores) at the 3 locations of the bias found in experiment 1, the leftward, upward, and central locations, and submitted the individual subjects’ values to a 2-way repeated measures ANOVA with factors *σ* (5, 10, 15°) and location (leftward, upward, and central). The results of the ANOVA revealed a main effect of *σ* (*F*(2,54) = 17.70, *p* < 0.001, ${\eta }_{p}^{2}$ = 0.396), a main effect of location (*F*(2,54) = 8.08, *p* = 0.001, ${\eta }_{p}^{2}$ = 0.230), and a significant interaction between the 2 (*F*(4,108) = 5.12, *p* = 0.001, ${\eta }_{p}^{2}$ = 0.160), consistent with a more pronounced bias towards the central compared to the upward and leftward locations, which was also more affected by changes in σ (see Fig. 6C). To obtain a broader picture of changes in the anisotropy of ensemble perception as a function of orientation variability, we performed an additional analysis in which a linear model predicted changes in the weight at each location as a function of *σ*. Confirming the ANOVA results, this revealed a significant increase in weight towards the Gabor at the central location (*p*_corr_ < 0.05, permutations statistics) but no changes at the other locations (all *p*s > 0.05, see Fig. 6B).

#### Effect of the ensemble duration

**Fig. 7:**
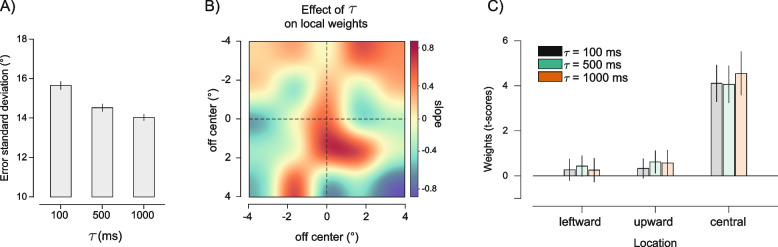
Results of experiment 3. **A** Standard deviation of the errors as a function of the ensemble duration *τ*. **B** Effect of *τ* on local weight changes. **C** Weights (*t*-scores) estimated at each of the three locations of interest (leftward, upward, central), as a function of the ensemble *τ*

In experiment 3, 21 participants reproduced the average orientation of the ensemble under 3 durations of the stimulus *τ*: 100, 500, and 1000 ms. The standard deviation of adjustment errors was inversely related to the stimulus duration: responses were more precise with longer stimulus presentations (Fig. 7A, F(2,40) = 18.269, *p* < 0.001, ${\eta }_{p}^{2}$ = 0. 477). As in experiment 2, we focused on changes in the weights at the 3 relevant locations as a function of the stimulus duration. A repeated measures ANOVA with factors duration (100, 500, and 1000 ms) and location (leftward, upward, and central) revealed only a main effect of location (*F*(2,40) = 12.90, *p* < 0.001, ${\eta }_{p}^{2}$ = 0.392, see Fig. 7C), with no effect of duration and no interaction (both *p*s > 0.05). In particular, spatial weights exhibited a strong bias towards the center, which was evident and comparable across all duration conditions (Additional file 1: Fig. S1). In line with this, a linear model predicting weights at each location as a function of the ensemble duration revealed no significant changes at none of the locations (all *p*s > 0.05). Importantly, spatial weights estimated in the 100 ms duration condition reliably predicted single-trial reproduction biases in the other two duration conditions. This suggests stable spatial biases unaffected by factors such as eye movements, which might be facilitated during longer stimulus durations (Additional file 1: Fig. S2).

Hence, while the ensemble variability strongly modulated the bias towards the center (experiment 2), the manipulation of stimulus duration did not affect the magnitude of the bias, which remained the same regardless of whether the stimulus lasted 100 ms, 500 ms, or 1000 ms (Fig. 7B).

The results of these two experiments indicated a dissociation between uncertainty, performance, and central anisotropy: while the variability in the ensemble’s orientations affected both averaging precision and spatial biases, the ensemble duration affected precision but not spatial biases. This suggests that, while increasing under uncertainty, the bias towards the center is a rather stable and persistent component of ensemble perception, even in conditions where the stimulus remains on screen for 1 s.

### Experiment 4

The first three experiments demonstrated that orientation ensemble statistics are biased towards the center of the visual field and nearest leftward and upward locations (experiment 1). The bias towards the center strongly increased with stimulus uncertainty but was immune to stimulus duration and was always the most prominent (experiments 2 and 3). Conversely, the leftward and upward biases were not affected by uncertainty to the same degree. Based on these results, we reasoned that the central bias and the leftward and upward ones may arise from different sources.

In principle, spatial anisotropies in ensemble perception might arise from transitory spatial biases, due to momentary shifts in the allocation of processing resources, or from the persistent accumulation of visual information from preferential regions of the visual field [43]. In experiment 4, to disentangle the nature of the anisotropies found in our experiments, we turned to a novel paradigm. Twenty-eight participants were presented with the same ensemble of Gabors as in experiment 1, but the temporal onset of each stimulus was jittered and randomized. Hence, observers could only represent the mean of the entire ensemble at the end of the sequence. If a spatial bias has a transitory nature, it can only be observed when, at the moment in which the bias is supposedly larger, there is a stimulus at the biased location. For instance, if observers are biased towards the left, but only at the beginning of the trial, the bias would become evident only in trials where the element on the left is shown at the beginning. Alternatively, if observers exhibit a steady bias towards one location, which is independent of the moment in time, the bias would be evident no matter the serial position of the elements at the biased location. By this logic, in this experiment, we investigated the time course of the leftward, upward, and central bias.

**Fig. 8:**
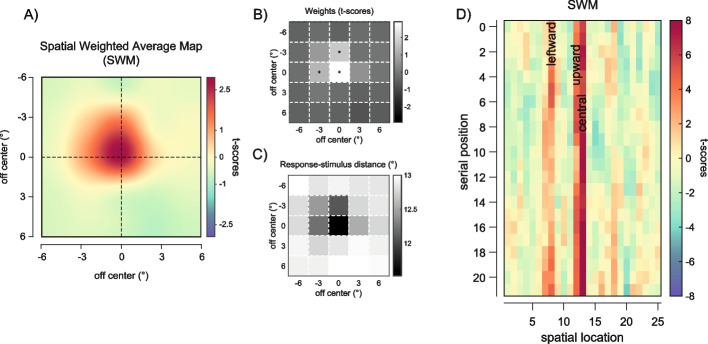
Results of experiment 4. **A** Spatial weighted maps (SWMs) represented as interpolated heat maps (see the “Methods” section). **B**, **C** Raw weights (*t*-scores) and the response-stimulus distance metric. The patterns in **A**–**C** are a clear replication of the results of experiment 1. **D** Matrix of weights (*t*-scores) at each location (columns) as a function of the serial position of each element in the sequence (rows). For example, the value in the first row, first column corresponds to the weight of the Gabor at the first location (top-left location, coded as 1) in trials where the stimulus at the top-left location appeared in the first frame of the sequence. The three locations of interest (leftward, coded as 8; upward, coded as 12; and central, coded as 13) are highlighted by their respective names

Overall, and independently of the serial position, the SWM showed anisotropies that were completely comparable to those observed in experiment 1, with significant biases towards the same three locations (leftward, upward, central location: *p*_corr_ < 0.05, all other *p*s > 0.05, see Fig. 8A, B). As in experiment 1, the results of the spatial weighted average model were confirmed by the response-stimulus distance analysis (Fig. 8C; all *d′* of the difference between each of the three locations and the remaining locations > 1).

**Fig. 9:**
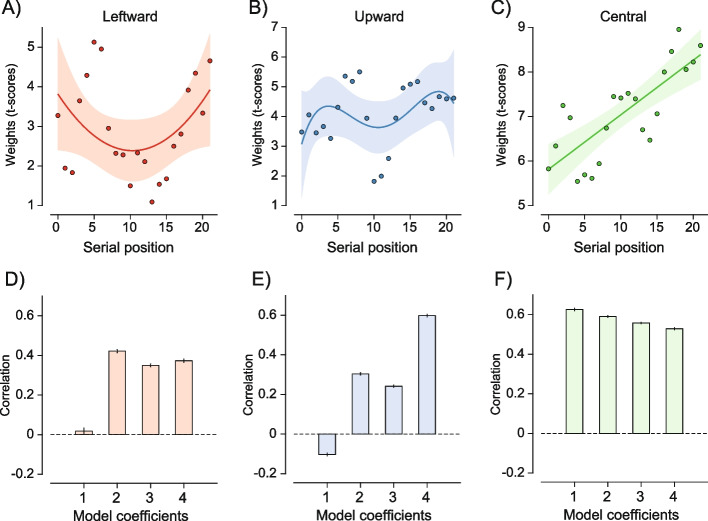
**A**–**C** Weights (*t*-scores) for the three locations of interest as a function of the serial position of the stimulus at the corresponding location. Dots are the weights estimated for each serial position. Prediction lines and shaded confidence intervals are obtained from the best-fitting model of each bias. Train-test correlation metric to evaluate the fit of polynomial models of increasing order for the time course of the leftward (**D**), upward (**E**), and central (**F**) bias

To investigate the temporal dynamics of the bias at the three locations of interest, we estimated SWMs and extracted the weight of the stimulus at each location depending on its serial position in the sequence, focusing on the central, leftward, and upward locations (Fig. 8D). By extracting the weights (*t*-scores) as a function of serial position, we derived the time course of each bias, corresponding to the possible serial positions of each stimulus in the sequence. To test for dynamic changes in the magnitude of each bias, we submitted the time course of the biases to model comparison (see the “Methods” section), comparing the fit obtained by models of increasing complexity. The model comparison revealed patterns that were clearly different between locations. By fitting the time course of each bias with polynomial functions of increasing order, we found that the bias towards the center was best approximated by a first-order polynomial (e.g., a linear function) with a positive slope, indicating an increase in the bias as the central stimulus appeared later in the sequence (Fig. 9C, F). Conversely, both the leftward and upward biases were better approximated by higher order polynomial functions (e.g., 4th or 2nd order), indicating more complex temporal dynamics, mostly characterized by increases in the bias when the stimulus at these locations appeared at the beginning and end of the sequence (Fig. 9A, B, D, E; see the “Methods” section). Hence, the two biases towards the leftward and upward locations had a more transitory component, whereas the central bias was stable and increased over time.

## Discussion

We used a weighted average model to recover the spatial weights with which observers extract the average orientation from an ensemble of visual stimuli. To investigate the relationship between spatial biases and uncertainty, we examined spatial biases as a function of the oblique effect, ensemble variability, stimulus duration, and temporal variability in element onset. The results of our four experiments reveal clear anisotropies in orientation averaging, with elements presented at the fovea and nearest locations contributing significantly more than what would be expected from a uniform averaging.

In all experiments, we found a robust bias towards the element shown at the fovea, which became more prominent as the level of uncertainty increased and persisted across different stimulus durations. Additionally, we also found a leftward and upward bias, although these biases showed less sensitivity to uncertainty and had more transient characteristics. The central bias, consistently observed across all four experiments and under various conditions, was the most robust and enduring bias. The leftward and upward biases were significant in only two out of the four experiments. These differences seem to indicate that the central and the leftward/upward biases have distinct origins.

The central bias can potentially be explained by the spatial arrangement of the elements within our displays. The central element was always placed at the foveal region, where receptive fields are narrower, and acuity is higher [31–33]. Thus, this systematic bias may simply reflect the greater weighting of foveal stimuli due to the larger number of local feature detectors. Even though ensemble statistics can be derived successfully in the absence of foveal stimuli (e.g., for peripheral ensembles [25, 57]), foveal stimuli provide significantly much more information.

However, this explanation fails to account for the observed increase in the central bias with uncertainty. If the bias were solely due to an imbalance between local feature detectors in the fovea and parafovea, it would remain constant in magnitude regardless of the properties of the ensemble. Instead, the increase in central bias with uncertainty suggests that the visual system integrates information from both the fovea and the parafovea/periphery when estimating ensemble statistics, in a non-uniform way. As uncertainty increases, stimuli presented at the fovea receive greater weight due to their higher reliability, following principles typical of classic cue integration schemes [58–60]. This may be particularly evident in our paradigm, where the uncertainty of the element shown at the fovea remained constant and equivalent to that of a single stimulus. Conversely, the uncertainty in the parafovea varied based on ensemble parameters such as the average orientation (as observed in the oblique effect) and orientation variability. As the uncertainty of the ensemble increased, the relative reliability of the central stimulus became higher.

In our experiments, we presented ensembles within a limited region of the visual field, primarily the fovea and peri/parafoveal regions. Hence, one may wonder how these anisotropies manifest when considering larger eccentricities or ensembles covering broader areas of the visual field. Previous research has shown that these biases are independent of element dispersion and remain confined to the central regions whether elements are all near the fovea or spread throughout the visual field [43]. This suggests that spatial anisotropies are mostly restricted to foveal and nearby locations, whereas ensemble perception may operate uniformly for the rest of the visual field. Future research may elucidate whether the localized nature of these biases reflects cortical or retinal sources and whether it involves variations in macular structure and function in the parafoveal region.

Our results may appear inconsistent with a study that aimed at dissociating crowding and ensemble perception. In crowding, a crowded target is better recognized in the lower visual field due to improved spatial resolution and smaller pooling regions. However, the study found no such asymmetry in ensemble perception, with comparable performance in estimating ensemble statistics in the upper and lower visual fields [45]. In contrast, our study revealed an upward bias in two out of four experiments. There are key differences between our study and the one from Bulakowski and colleagues (2011). First, the authors measured the overall accuracy for ensembles shown on the upper or lower visual field while we used a spatial weighted average model that can reveal biases limited to one or a few locations. Second, they used larger regions for ensembles, while our biases were limited to foveal and parafoveal regions. Third, the whole ensemble in Bulakowski et al. was presented in the periphery, and thus, there was no stimulus in the locations of spatial biases observed in our study [45].

An additional aspect of our results is the relationship between exposure duration and the bias towards the center. In experiment 3, we found that the central bias remained constant regardless of the increasing duration of ensemble exposure. In experiment 4, we found that the bias increased as the central element occurred later in the stimulus stream. Previous work has shown that ensemble statistics can be extracted very rapidly, and the benefit of longer exposure might be only minimal [61]. Here, we found that exposure duration can increase the precision of ensemble statistics, without necessarily altering the spatial bias. The persistence of the central bias with exposure duration (experiment 3) and its increase with the serial position of the central element (experiment 4) seem to suggest that observers keep relying more on and awaiting evidence from the foveal region while estimating ensemble statistics. It is also worth considering that in experiment 3, the uncertainty associated with both foveal and parafoveal stimuli might have increased as the display duration decreased. Thus, we might not have observed an increase in central bias at shorter durations, simply because the uncertainty on the central element also increased. Likewise, the increase in central bias as a function of serial position in experiment 4 could be due to increased uncertainty when estimating ensemble statistics through sequential presentations, potentially leading to a greater reliance on the central item as the elements unfolded.

Unlike the central bias, the leftward and upward biases were not observed in all experiments. In particular, under high ensemble variability (experiment 2) and short exposure durations (experiment 3), the weights assigned to leftward and upward locations did not significantly differ from weights at other locations. The leftward and upward biases also did not exhibit an increase with increasing uncertainty (experiment 2) and did not persist or increase with exposure duration and serial position, but rather showed patterns resembling primacy and recency effects (Fig. 9A, B). It is possible that the sources of the leftward and upward biases differ from the central bias and may be potentially related to the temporal distribution of spatial attention and scanning patterns [49, 62, 63]. As suggested by prior work [43, 44], a potential cause of the leftward bias is the reading habits, since most of our participants read from left to right and up to down [64, 65]. Thus, although speculative, the temporal rise and fall of these biases observed in experiment 4 (Fig. 9) may reflect initial “hard-wired” transitory biases in spatial processing, followed by inhibition and subsequent re-engagement with similar biases [66–68].

One potential limitation of our study is the absence of eye movement recordings and the use of a large placeholder frame to indicate the start of each trial instead of a traditional fixation spot. However, several factors suggest that the impact of eye movements on our results is likely minimal. Firstly, prior research has demonstrated that individuals tend to direct their fovea towards the center of mass when confronted with large or multiple objects [69–71], which is consistent with our central item in the ensemble. Secondly, the spatial weighted average maps we observed closely resemble those found in previous experiments involving size averaging, where fixation was indicated by a clearly defined central spot, and stimulus duration was shorter [43]. Thirdly, any central bias resulting from systematic eye movements would imply fixation at the center. Lastly, the supplementary analysis involving a cross-validation approach (Additional file 1: Fig. S2) demonstrates that one can use spatial anisotropies estimated in the 100 ms condition to reliably predict single-trial errors in the 100 ms, 500 ms, and 1000 ms conditions, and thus, any potential influence of eye movements during longer stimuli presentations does not significantly alter the observed spatial biases.

Conversely, we cannot rule out that the leftward and upward biases, rather than stemming from distinct sources, may still reflect the central bias in trials where participants systematically foveated on those locations. This is supported by the observed maps, which align with patterns seen in prior research on initial saccade landing points during free viewing, where most saccades target the center and the left side [43, 49]. Future research is needed to clarify the precise interplay between eye movements and the leftward/upward bias found in the present study.

From a neural perspective, the central bias can be explained through sequential uncertainty-weighted pooling stages. Initially, visual features are pooled over local regions, such as the fovea and parafovea, followed by a second pooling stage where the resulting estimates are weighted and combined based on their relative reliability [72, 73]. These findings hold significant importance for ongoing efforts in modeling ensemble perception, as existing models fail to account for these anisotropies, their temporal dynamics, and their computational goals. Spatially pooling models, for example, typically assume a uniform pooling [24, 26]. Other models that incorporate local pooling with regions that increase in size with eccentricity, such as image statistics models [11, 19], do not consider local uncertainty. Similarly, alternative models based on sub-sampling, where statistics are derived from a small randomly selected subset of items. [27–30], fail to account for the influence of the whole ensemble uncertainty on the magnitude of the bias. Thus, existing models must incorporate an additional component, whose implementation may vary depending on the model, to account for the highly spatially anisotropic and uncertainty-weighted nature of ensemble perception. It is important to clarify that while certain regions are given more weight, it does not imply that information from other regions is completely disregarded, nor does it suggest that statistics cannot be accurately derived in the absence of elements at or near the fovea [25, 57].

## Conclusions

In sum, our findings highlight the importance of considering spatial anisotropies and incorporating uncertainty-weighted mechanisms in ensemble perception. The brain integrates multiple elements through adaptive compensatory strategies, assigning greater weights to more reliable regions of the visual field, in order to mitigate the impact of visual noise and uncertainty. These findings emphasize the need for all models of ensemble perception to incorporate these factors, as they play a crucial role in optimizing the efficiency of ensemble perception.

## Methods

### Participants

In total, 127 participants (92 after exclusion) participated in the experiments. Experiments 1 and 4 were conducted in the laboratory and included 44 participants (15 in experiment 1, 7 females; age range 19–30; 29 in experiment 4 (28 after exclusion), 9 females; age range 18–30) from the EPFL and the University of Lausanne, who participated for monetary reward (25 CHF/h). Experiments 2 and 3 were performed online and included 83 participants, 49 participants after exclusion (40 in experiment 2 (28 after exclusion), 11 females; age range 18–40; 43 in experiment 3 (21 after exclusion), 23 females; age range 18–39), recruited through the Prolific platform (www.prolific.ac) [74, 75] and Pavlovia (https://pavlovia.org), with a monetary reward of £5/h. All participants reported normal or corrected-to-normal vision and were naïve as to the purpose of the experiments. In laboratory experiments, visual acuity was assessed using the Freiburg Visual Acuity Test and a threshold of 1 for inclusion (Freiburg Visual Acuity Test) [76]. The study was approved by the local ethics committee following the Declaration of Helsinki. Written informed consent was obtained from each participant before the experiment.

### Apparatus

The stimuli in experiment 1 were presented on a VG248QE monitor (diagonal 24″, resolution 1920 × 1080 pixels, refresh rate 120 Hz), whereas in experiment 4, they were presented on a BenQ XL2420T (24″, 1920 × 1080, 120 Hz). For both experiments 1 and 4, the stimuli were generated with the Psychophysics Toolbox 3.8 on MATLAB [77, 78] version 3.1, 64 bits R2014b. Laboratory experiments were performed in a dimly lit room, and participants sat 57 cm away from the computer screen.

The stimuli in experiments 2 and 3 were generated and presented online via PsychoPy v2020.2.10 [79, 80]. Before the experiment, all participants completed a virtual chin-rest procedure [81] consisting of (1) participants adjusted an object of known size (a bank card) shown on the screen to match their physical size and (2) the program measured the horizontal distance from fixation to the blind spot on the screen to estimate the viewing distance and calibrate the screen pixels per degree.

### Stimuli and task procedure

In all experiments, the stimuli were ensembles of Gabor patches (peak contrast of 25% Michelson, spatial frequency of 2 cycles per degree) arranged on an invisible grid of 5 × 5 cells (size of the grid in experiments 1, 2, and 3: 8 × 8°; experiment 4: 12 × 12°). The grid was surrounded by an empty green square frame (experiments 1, 2, and 3: 12 × 12°; experiment 4: 14 × 14°). Each Gabor patch in the ensemble was presented in the middle of each cell of the grid with a random horizontal and vertical jitter (± 0.25°) and with the constraint that two nearby Gabor patches never overlapped. The standard deviation of the Gaussian envelope determining the size of each Gabor was equal to 1.5° in experiments 1 and 4 and 0.7° in experiments 2 and 3.

The average orientation of the Gabor ensemble was randomly determined on each trial by sampling from the entire orientation space (in experiment 1, the 0–180° space was discretized, in steps of 10°, in all the other experiments the steps were of 1°). The standard deviation of the ensemble distribution was 10° in experiments 1, 3, and 4, whereas it randomly varied between 5, 10, and 15 in experiment 2, following different experimental conditions. The response tool, used in all experiments, was made of two dark gray circles connected by an imaginary line. To avoid any systematic focus on the center of the monitor throughout the experiments, the response tool appeared at random locations inside the green frame.

The procedure of all four experiments was similar, except for experiment 4 (see the “Methods” section). The trial started with the empty green square frame (1000 ms) and was followed by the ensemble of Gabor patches appearing inside the frame. The duration of the ensemble presentation was 500 ms in experiments 1 and 2 and 100 ms, 500 ms, or 1000 ms in experiment 3, correspondingly to the experimental conditions. After a blank interval (500 ms in experiments 1; 1000 ms in experiments 2 and 3), the response tool appeared. Participants were asked to adjust the tool to the perceived average orientation of the ensemble. The orientation adjustment was performed by moving the computer mouse upward (to tilt clockwise) or downward (to tilt counterclockwise). To confirm the response, participants had to click the left button of the mouse.

In experiment 4, each trial started with the empty green square frame (1000 ms), and then the ensemble of Gabor patches appeared inside the green frame item by item. Each Gabor patch appeared for 300 ms, and the order of appearance (serial position) was randomized across trials. No constraints were imposed, such that two Gabor patches could appear at the same time or with any possible interval, within the time window of one trial. The total duration of the ensemble presentation—from the appearance of the first Gabor patch to the disappearance of the last—was 800 ms. As in the other experiments, after a blank interval (500 ms), participants were asked to report the perceived average orientation of the ensemble using the response tool.

Participants performed a brief practice session before each experiment and were instructed to maintain fixation on the center of the placeholder frame, while paying attention to all the elements in the display. Each experiment lasted approximately 1 h. Note that, even though the placeholder frame may not be as effective as classic central cues to maintain fixation, the observed spatial biases were completely comparable to those found in previous work using more canonical fixation points [43].

### Data analysis

Before the analysis, adjustment responses were cleaned from outliers, by removing errors (the acute angle between the reported and true average orientation) larger than 45° and then removing additional outliers identified as values more than 1.5 interquartile ranges above the upper quartile or below the lower quartile. Responses slower than 10 s were also removed. In total, less than 6% of outlier trials were removed. The datasets of this article are available in the Zenodo repository [82].

In total, the data of 35 participants were excluded from the final analysis (12 in experiment 2, 22 in experiment 3, and 1 in experiment 4) according to the following exclusion criterion: a standard deviation of errors higher than 30°.

To estimate the spatial weighted average model (SWM), we adapted a method previously used in the investigation of size averaging [43]. Because of the circular orientation variables, we transformed the response into “error” and the orientation of each Gabor patch in the ensemble into “deviation.” This transformation involved computing the acute angle difference between the variable of interest (e.g., the reported orientation, or the orientation of each Gabor) and the true average orientation presented on each trial. The transformed variables approximated a general normal distribution centered approximately on 0°.

The estimation of SWM involved solving an ordinary least squares regression (using the Moore Penrose pseudo-inverse) to recover the linear weights with which the “deviation” of each Gabor at each location contributed to the error made on each trial. This was obtained via the following model:

$${\varepsilon }_{ij}=\sum_{k=1}^{n}{w}_{k}{\delta }_{jk}$$

where ${\varepsilon }_{ij}$ is the response error of observer $i$ on trial $j$, ${w}_{k}$ is the weight of the $k$ th of $n$ elements in the ensemble (with $n$ = 25), and ${\delta }_{jk}$ is the deviation of the $k$ element on trial $j$ from the actual average orientation of the ensemble. Weights were estimated for each observer and standardized across observers via transformation into regression *t*-scores. For some of the analysis, we report a measure of “bias” rather than weights, computed by subtracting the weight at the location of interest from the weights at other control locations (see the description for each experiment in the “Result” section).

To control that the estimated weights (*t*-scores) were not artifactual or biased by the linear approximation of circular data, in experiments 1 and 4, we also verified the results using a measure called the “response-stimulus distance,” which corresponds to the absolute deviation of the response from the orientation of the Gabor at each location. The SWM and the response-stimulus distance results should mirror each other: a location receiving larger weights is also a location where the error tends to be more similar to the Gabor, and the distance of the report from the Gabor orientation is lower.

For significance testing, we used a non-parametric permutation approach at the group level. The correspondence between weights and locations at the level of individual observers was permuted 10,000 times via random shuffling (e.g., disrupting any relation between the estimated weight and the location across participants). For each permutation, we computed the group average of weights at each location, obtaining a surrogate null distribution of weights assuming no relationship with spatial locations. We then calculated the proportion of permutations in which the surrogate weight at each location was larger than the true estimated average weight, thresholding the resulting *p*-value with a Bonferroni-corrected alpha criterion of 0.05. Heat maps were obtained via linear interpolation of the original weights matrix.

In experiments 2 and 3, ensembles were presented under different conditions of increasing uncertainty (experiment 2) and increasing duration (experiment 3). To investigate the effects of these factors, we used a two-stage approach. First, individual SWMs were estimated for each condition and participant. Statistical testing of SWM for each condition was performed according to the same permutation procedure as in experiment 1. Then, individual weights maps were submitted to linear modeling at the group stage, where the weights corresponding to the Gabor patch at each location were predicted using the levels of the variables of interest (e.g., the three standard deviations of the ensemble used in experiment 2; the three durations used in experiment 3). The resulting group map of coefficients from this second model quantified the increase or decrease of the bias towards each Gabor, depending on variations in the variable of interest. Statistical testing of the coefficients was performed according to a similar permutation procedure as the one described above.

In experiment 4, we aimed at reconstructing the time course of SWMs as a function of the serial position of each Gabor in the sequence. To this aim, we combined the data across participants, after *z*-scoring the individual distribution of errors. From the aggregate dataset, we estimated multiple independent SWMs considering the serial position of the Gabor at each spatial location (from 1 to 25 locations), with a moving window of 3 serial positions each time (leading to a series of estimates from 1 to 22 serial positions). The SWM for each Gabor location was solely based on the serial position of that Gabor relative to the others, whereas the serial position of all the others could vary. From the SWM of each Gabor, we selected the weights estimated for the effect of that Gabor as a function of its serial position and combined them in an overall matrix (Gabor location X serial position, Fig. 8D). This matrix describes the changes in weights for each Gabor depending on its serial position in the sequence. Focusing on the central, leftward, and upward locations, we tested for changes in weights across time (e.g., serial position) by means of model comparison. The pattern of weights over time was fitted with a series of polynomials of increasing order (from 1 to 4), accounting for simple linear trends and more complex temporal dynamics. Model comparison was performed via cross-validation, randomly splitting the participants in half 10,000 times, and using half as the training set and the other half as the test set. The training set was used to fit the set of models. The correlation between the predictions of each model and the unseen data in the test set was then used as a metric of relative model performance.

### Supplementary Information

**Additional file 1.** Supplementary analysis of Experiment 3. **Fig. S1.** Grayscale representation of raw weights (model coefficients) from the spatial weighted average model for the three duration conditions of Experiment 3. The spatial anisotropies, notably the central bias, exhibit stability and consistency across different stimulus durations, with white indicating larger weights. **Fig. S2.** Model results using spatial anisotropy from the 100 ms condition (training) to predict single-trial errors in the 100 ms, 500 ms, and 1000 ms conditions (tests) through cross-validation (5-fold). Each individual subject is represented by open blue, red, and green circles for the 100, 500, and 1000 ms conditions, respectively, with evaluation based on the correlation between predicted and observed single-trial errors in the left-out data of the same subject (averaging cross-validation folds). This supplementary analysis aims to determine if variance in longer duration conditions can be explained by anisotropy in the 100 ms condition, indicating minimal influence of differential eye movements due to longer stimulus durations on the results. The average model performance remains consistent across duration conditions, significantly exceeding chance levels (open gray circles and bars, illustrating the median and upper and lower 99th quantiles of correlations obtained by shuffling observed errors across trials). Consequently, spatial maps derived from the 100 ms stimulus duration demonstrate stability and consistency as stimulus duration increases, and exhibit the ability to predict single-trial errors at other durations with the same model performance.

## Data Availability

All data generated or analyzed during this study are included in this published article and its supplementary information files. The datasets supporting the conclusions of this article are available in the Zenodo repository, 10.5281/zenodo.10406375. Code can be accessed upon request.

## Abbreviation

SWM: Spatial weighted maps
