# Supplementary material for: The functional role of spatial anisotropies in ensemble perception
Source: BMC Biol. 2024 Feb 5;22:28. doi: 10.1186/s12915-024-01822-3 (PMC10845794; doi:10.1186/s12915-024-01822-3)
Supplement: Supplementary file 1 — Additional file 1. Supplementary analysis of Experiment 3. Fig. S1. Grayscale representation of raw weights (model coefficients) from the spatial weighted average model for the three duration conditions of Experiment 3. The spatial anisotropies, notably the central bias, exhibit stability and consistency across different stimulus durations, with white indicating larger weights. Fig. S2. Model results using spatial anisotropy from the 100 ms condition (training) to predict single-trial errors in the 100 ms, 500 ms, and 1000 ms conditions (tests) through cross-validation (5-fold). Each individual subject is represented by open blue, red, and green circles for the 100, 500, and 1000 ms conditions, respectively, with evaluation based on the correlation between predicted and observed single-trial errors in the left-out data of the same subject (averaging cross-validation folds). This supplementary analysis aims to determine if variance in longer duration conditions can be explained by anisotropy in the 100 ms condition, indicating minimal influence of differential eye movements due to longer stimulus durations on the results. The average model performance remains consistent across duration conditions, significantly exceeding chance levels (open gray circles and bars, illustrating the median and upper and lower 99th quantiles of correlations obtained by shuffling observed errors across trials). Consequently, spatial maps derived from the 100 ms stimulus duration demonstrate stability and consistency as stimulus duration increases, and exhibit the ability to predict single-trial errors at other durations with the same model performance. [file 12915_2024_1822_MOESM1_ESM.docx]

# Supplementary Material:

**The functional role of spatial anisotropies in ensemble perception**

Authors: *Tiurina Natalia^1,2^, Markov Yuri^1,3^, Whitney David^4,5,6^, Pascucci David^1^*

1-Laboratory of Psychophysics, Brain Mind Institute, École Polytechnique Fédérale de Lausanne (EPFL), Switzerland

2-Department of Psychology, Technische Universität Dresden, Dresden, Germany

3-Department of Psychology, Goethe University Frankfurt, Frankfurt am Main, Germany

4-Vision Science Graduate Group, University of California, Berkeley, Berkeley, USA

5-Department of Psychology, University of California, Berkeley, Berkeley, USA

6-Helen Wills Neuroscience Institute, University of California, Berkeley, Berkeley, USA

#
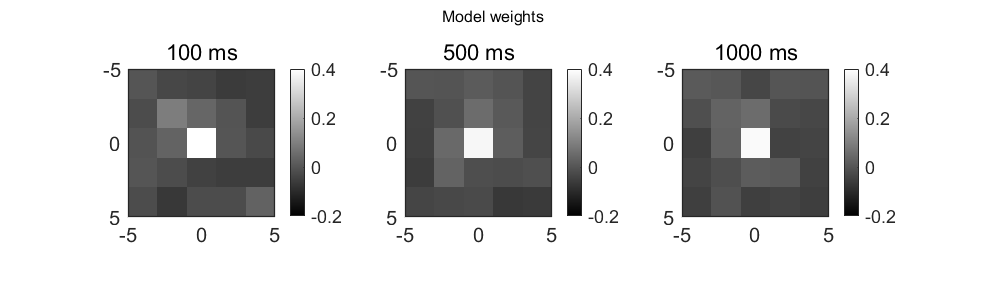
Figure S1

Grayscale representation of raw weights (model coefficients) from the spatial weighted average model for the three duration conditions of Experiment 3. The spatial anisotropies, notably the central bias, exhibit stability and consistency across different stimulus durations, with white indicating larger weights.

#
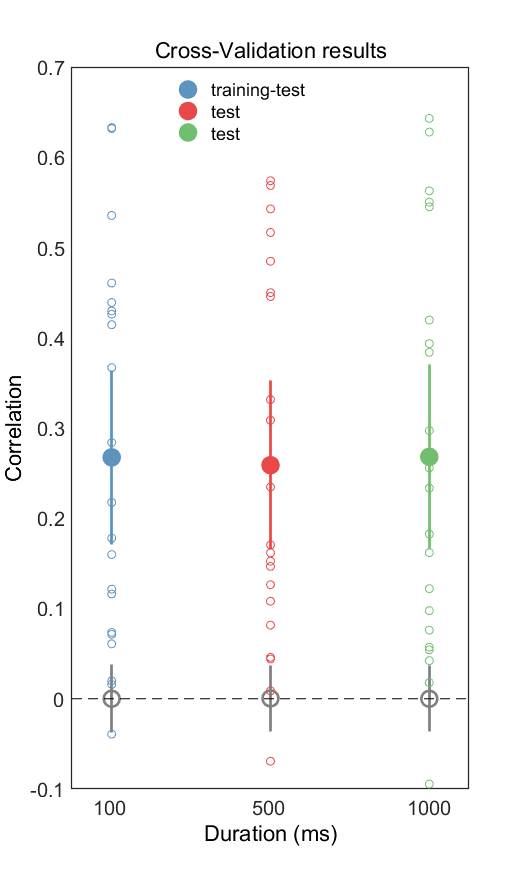
Figure S2

Model results using spatial anisotropy from the 100 ms condition (training) to predict single-trial errors in the 100 ms, 500 ms, and 1000 ms conditions (tests) through cross-validation (5-fold). Each individual subject is represented by open blue, red, and green circles for the 100, 500, and 1000 ms conditions, respectively, with evaluation based on the correlation between predicted and observed single-trial errors in the left-out data of the same subject (averaging cross-validation folds). This supplementary analysis aims to determine if variance in longer duration conditions can be explained by anisotropy in the 100 ms condition, indicating minimal influence of differential eye movements due to longer stimulus durations on the results. The average model performance remains consistent across duration conditions, significantly exceeding chance levels (open gray circles and bars, illustrating the median and upper and lower 99th quantiles of correlations obtained by shuffling observed errors across trials). Consequently, spatial maps derived from the 100 ms stimulus duration demonstrate stability and consistency as stimulus duration increases, and exhibit the ability to predict single-trial errors at other durations with the same model performance.
